# Potentially Toxic Element Contamination in Soils Affected by the Antimony Mine Spill in Northwest China

**DOI:** 10.3390/toxics11040359

**Published:** 2023-04-10

**Authors:** Yongzhen Chai, Fei Guo

**Affiliations:** State Key Laboratory of Environmental Criteria and Risk Assessment, Chinese Research Academy of Environmental Sciences, Beijing 100012, China; chaiyongzhen2021@163.com

**Keywords:** mining spill, potentially toxic elements, soil pollution, risk assessment, multivariate analysis, antimony

## Abstract

This study provides a comprehensive assessment of the potential ecological and health risks in the area of the antimony mine spill in Longnan, Northwest China, and identifies the sources of potentially toxic elements (PTEs) in the soil as a result of the spill. The geo-accumulation index and enrichment factor show that the study area is highly contaminated with arsenic (As), mercury (Hg) and antimony (Sb). The ecological risk index in the tailings spill area ranged from 320.43 to 5820.46 (mean: 1489.82), indicating a very-high potential ecological risk, with mean values of 104.86, 1118.87 and 248.84 for As, Hg and Sb, respectively. The multivariate statistical analysis suggested that Sb and Hg come from tailings leakage, while copper (Cu), nickel (Ni) and zinc (Zn) may be imported from natural sources, and As and lead (Pb) originate from agricultural activities. In addition As and Sb pose a high health risk. With the exception of the non-carcinogenic risk in adults, all other risks are significantly exceeded in other populations, with children being the highest-risk group. These findings provide important quantitative information for the assessment and management of PTE contamination in other tailings spill areas.

## 1. Introduction

Tailings from mining activities worldwide have adverse effects on both the environment and human health [1,2,3]. In addition, the negative impacts remain even in mines that were previously mined and nearby soils may have high levels of potentially toxic elements (PTEs) [4,5]. Previous studies have demonstrated that metal mining and smelting could lead to a higher concentration of PTEs in surrounding areas [6,7,8]. The generation of tailings and wastewater during the mining and smelting process also has the potential to cause more serious consequences, such as tailings overflow accidents. In 2015, the largest tailings leakage accident occurred in Minas Gerais, Brazil, which resulted in the leakage of more than 35 million cubic metres of slag, 19 deaths and river pollution affecting and area of more than 650 Km [9].

Contamination of soil with PTEs is a non-naturally degradable, persistent and untransformable problem [10,11] that reduces the quality of air, water and food crops. As PTEs move through the food chain, they threaten the health of animals and humans [12]. For instance, antimony (Sb) and its compounds can cause antimony poisoning, leading to damage to various organs and tissues, including the nervous system [13]. In recent years, adverse effects on crops and local farmers caused by antimony and arsenic (As) have been reported [14], and pollution involving different PTEs has damaged ecosystems and endangered the health of the population to some extent [15,16]. In China, on 23 November 2015, an antimony tailings reservoir leaked near the Taishi River in the Jialing River basin. Contamination stemming from tailings flowed into Gansu, Shanxi and Sichuan provinces along the Jialing River, causing serious environmental pollution in these areas. Moreover, emergency soil removal procedures were implemented immediately after the accident to mechanically remove toxic sludge from the tailings spill area. However, a proportion of the contaminated farmland near the accident site still remains polluted by tailings sand. Since the contamination of agricultural soils with PTEs is directly related to human health and safety, the environmental risks caused by this accident deserve attention.

The ecological and health risks associated with PTEs in soil surrounding mining sites necessitate risk assessment. Several methods, including the geo-accumulation index (*I_geo_*), enrichment factor and potential ecological risk assessment have been used to assess the ecological risks of PTEs in soil [17,18,19]. Multivariate analysis techniques, such as the Pearson correlation analysis, cluster analysis and principal component analysis (PCA), can be employed to determine the sources of PTEs in soil and allocate natural and human contributions [12,20,21]. The most classic approach to human health risk evaluation is to use the health risk assessment model recommended by the United States Environmental Protection Agency (USEPA). PTEs in soil can pose non-carcinogenic and carcinogenic risks to humans through ingestion, inhalation, and dermal contact [22]. A more detailed and reasonable assessment can be achieved by dividing the assessment subjects into three different age groups: children from 0 to 5 years old, adolescents from 6 to 17 years old and adults aged 18 years old and above.

Therefore, the purposes of this study were: (1) to determine the concentration of PTEs in the soil surrounding the tailings spill area; (2) to assess the ecological risks of PTEs in soil; (3) to qualitatively determine the main sources of PTE contamination in soil through multivariate analysis; and (4) to assess the human health risk caused by PTEs in soil. The findings of this study could provide valuable insights into the environmental risks associated with PTEs in soil and inform risk management strategies.

## 2. Materials and Methods

### 2.1. Study Area and Sampling Spots

**Figure 1:**
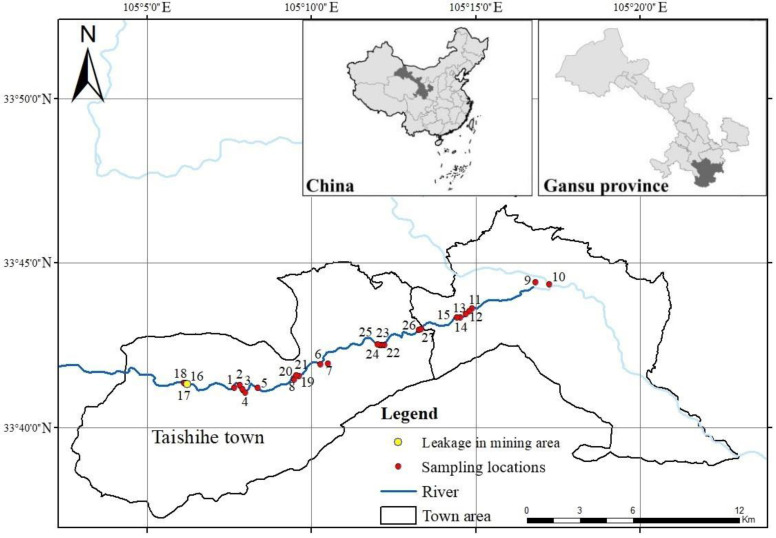
Map of the study area and sampling locations. The numbers on the figure indicate the point numbers of the sampling locations. There are 27 sampling points.

The mineral resources of Xihe County are abundant, including lead (Pb), zinc (Zn), Sb, copper (Cu), iron, gold, silver and other metallic minerals. This area is the westward extension of the second-largest lead-zinc ore belt in the China-Xicheng Lead-Zinc Ore Belt [23]. The study area encompassed an antimony mine in Xihe County (105.3° E, 34.02° N) (Figure 1). At approximately 21:20 on 23 November 2015, the drainage well of the tailings pond in the mining area was damaged, resulting in a large amount of tailings and tailings water flowing into the drainage well through the hole and then through the drainage pipe into the drainage culvert. This resulted in the tailings and tailings water gushing out from the culvert hole and into the adjacent Taishi River (a tributary of the Xihan River, the first branch of the Jialing River) [24]. Approximately 25,000 m^3^ of tailings and tailings water leaked from the tailings pond [25]. After the accident, mine waste was discharged into the lower reaches of the Taishi River, which is mainly used for the irrigation of crops, causing serious damage to the surrounding environment. Figure 1 shows the 27 agricultural soil sampling sites along the Taishi River.

### 2.2. Sample Collection and Analysis

Soil samples for this study were collected from 0 to 20 cm of topsoil and three subsamples were randomly collected at each sampling point and combined into one composite sample after quadratting. The collected samples were stored in pre-prepared polyethylene bags and promptly sent to the laboratory. Soil samples were air-dried and ground until they all passed through a 0.2 mm pore size nylon sieve. Then, they were packed into polyethylene bags for subsequent analysis. The soil collection and analysis methods for this study were carried out in accordance with the relevant technical requirements of the Chinese Technical Specification for Soil Environmental Monitoring (HJ/T166–2004) [26].

The samples were digested in Teflon tubes with HNO_3_, HCl, HF and HClO_4_ to determine the contents of As, Cu, mercury (Hg), nickel (Ni), Pb, Sb and Zn. The contents of Sb, As and Hg in the samples were determined via atomic fluorescence spectrometry (AFS), and the contents of Cu, Ni, Pb and Zn were determined via flame atomic absorption spectrometry (FAAS). To ensure the accuracy of the experiments, the Chinese standardized reference materials (GSS-1) provided by the Chinese National Research Center for Standard Reference Materials were used for validation. Twenty percent of the soil samples were randomly selected for replicate measurements, and the standard deviation (S.D.) of the replicate samples was less than 4%. Blank samples were measured to maintain the experimental accuracy when measuring the content of each PTE. The detection limits (AFS) for As, Sb and Hg were 0.05, 0.05 and 0.002 mg/kg, respectively, and the detection limits (FAAS) for Cu, Ni, Pb and Zn were 0.002, 0.004, 0.02 and 0.002 mg/kg, respectively. The instrument was calibrated using standard substance solutions produced by the National Institute of Metrology, China, and the specific standard substances are listed in Table S1.

### 2.3. Assessment of PTEs Contamination

#### 2.3.1. Geo-Accumulation Index

A cumulative index was adopted to evaluate the degree of soil contamination by some PTEs in the tailings spill area. The geo-accumulation index (*I_geo_*) was originally proposed as a quantitative indicator of the extent of contamination of sediments with heavy metals and other substances [27].

(1) $$I_{\mathrm{geo}}=log_{2}\left(\frac{C_{n}}{1.5B_{n}}\right)$$

where *C_n_* was the measured concentration of potentially toxic element n (As, Cu, Hg, Ni, Pb, Sb and Zn) in the tested soil, *B_n_* was the geochemical background concentration in Gansu Province, and the factor 1.5 in Equation (1) was used to neutralize the effects caused by lithospheric effects. *I_geo_* was divided into seven classes to assess the degree of PTE contamination, and the specific classes are listed in Table 1 [28].

#### 2.3.2. Enrichment Factor

The *EF* was used to assess the extent of the effect of anthropogenic activities on the content of PTEs in the soil. To reduce any artificial influences on the test process and ensure comparability and equivalence among the indicators, the elements in the test samples were normalized with reference elements as the reference standards [31]. Reference elements are required to remain relatively stable and not be easily affected by the environment or analysis process. Common reference elements include Se, Mn, Al, Fe and Ca [32]. In this study, Al was selected as the reference element since Al is the most abundant metal element in the Earth’s crust and exhibits stable properties of a low solubility and bioavailability, which suggests difficulty in leaching and migration. The *EF* value was calculated using the equation given in the literature [33]:

(2) $$\mathrm{EF}=\frac{C_{N}}{C_{REF}}\times \frac{B_{REF}}{B_{N}}$$

In the above equation, the *EF* is the enrichment coefficient of the considered PTEs in soil, and *C_N_* and *C_REF_* are the concentrations of an element and the reference element in the sample of the study area, respectively. *B_N_* and *B_REF_* are the concentrations of an element and the reference element in the background area, respectively. According to the determined *EF* values, the PTE pollution degree was divided into five different levels (Table 1).

#### 2.3.3. Ecological Risk Assessment

Compared to *I_geo_* and other methods, the potential risk approach can be applied to assess the risk of contamination of various types of heavy metals across ecosystem portfolios [34]. Therefore, when studying the potential ecological risk that may be caused by the contamination of soil with PTEs, the potential ecological risk index method was used to combine the ecological environment with the toxicological effects of PTEs for assessment. In the current study of PTE contamination in soils in China, the potential ecological hazard index method was adopted [35,36,37]:

(3) $$C_{r}^{i}=C_{s}^{i}/C_{n}^{i}$$

(4) $$E_{r}^{i}=T_{r}^{i}\times C_{r}^{i}$$

(5) $$\mathrm{RI}=\sum_{i=1}^{n}E_{r}^{i}$$

The pollution factor of element *i* ($C_{r}^{i}$) was determined using Equation (3). $C_{s}^{i}$ is the measured content of the potentially toxic element *i* in the soil, and $C_{n}^{i}$ is the evaluation criterion of this element.

The potential ecological risk index of an individual PTE ($E_{r}^{i}$) was calculated using Equation (4), which describes the degree of contamination of a certain pollutant element, and it is divided into 5 levels in ascending order. The toxic response coefficient $T_{r}^{i}$ of each PTE reflects the toxic strength of the PTE and the sensitivity of a given water body to this PTE. The comprehensive potential ecological risk index (*RI*) was calculated using Equation (5), which describes the combined value of the potential ecological risk index for all elements at each sampling site, and it is divided into four classes, as summarized in Table 1.

According to previously published reports, the $T_{r}^{i}$ values of As, Cu, Hg, Ni, Pb and Zn are 10, 5, 40, 5, 5 and 1, respectively [30]. It is generally considered that Sb is similar to As in chemical behaviour and toxicity [38]. Therefore, the toxicity value of Sb in this study was taken to be 10.

### 2.4. Assessment of Human Health Risks

Health risk assessment is an evaluation method that considers the contaminant under study as a risk source and human health as a risk receptor and aims to explore whether human health is potentially threatened. The human health risk assessment system can derive the average daily dose (*ADD*) of an element entering the human body through certain exposure pathways from the known concentrations of PTEs, and thus obtain the non-carcinogenic risk faced by exposure to pollutants and the carcinogenic risk due to exposure to certain carcinogenic elements. This study divided the risk receptors into three different groups according to age: children (0–5 years), adolescents (6–17 years), and adults (18 years and older) in order to obtain more detailed results. There are three general exposure pathways to assess health risks due to PTE contamination in soil: ingestion, inhalation and dermal contact. The *ADD* of the PTE for each pathway was calculated as listed in the following Equations (6)–(8) [15,39]:

(6) $$ADD_{ing}=\frac{C_{s}\times IngR\times EF\times ED}{BW\times AT}\times 10^{-6}$$

(7) $$ADD_{inh}=\frac{C_{s}\times InhR\times EF\times ED}{PEF\times BW\times AT}$$

(8) $$ADD_{der}=\frac{C_{s}\times SA\times AF\times ABS\times EF\times ED}{BW\times AT}\times 10^{-6}$$

where *ADD_ing_*, *ADD_inh_* and *ADD_der_* are the mean average doses of PTEs by ingestion, inhalation and dermal contact, respectively (mg kg^−1^ day^−1^); *C_s_* is the concentration of PTE in the soil (mg kg^−1^). Other parameters related to human activities are listed in Table S2 [40,41,42,43,44,45].

The hazard quotient (*HQ*) calculated for each PTE can be summed to obtain a hazard index (*HI*), which was used to assess the non-carcinogenic risk to humans from the PTE contamination studied in the study area. This can be calculated as follows:

(9) $$HQ=\frac{ADD}{RfD}$$

(10) $$HI=\sum_{i=1}^{7}HQ$$

where RfD represents the reference doses of PTEs (mg kg^−1^ d^−1^).

If the value of *HQ* or *HI* > 1, PTE contamination may cause adverse effects on human health. If the value of *HQ* or *HI* ≤ 1, it indicates that the non-carcinogenic risk of PTEs is not significant [46,47].

The possible carcinogenic risk of individual PTEs with carcinogenic properties is quantified by the cancer risk (*CR*) value. The *CR* values of several elements are summed to obtain the cumulative carcinogenic risk (*CCR*) to human health in the region, calculated by Equations (11) and (12):

(11) CR=ADD×CSF

(12) $$CCR=\sum_{i=1}^{7}CR$$

where *CSF* represents the cancer slope factors of PTEs ((mg kg^−1^ d^−1^) ^−1^).

If the value of *CR* or *CCR* ≤ 10^−6^, the PTE contamination does not have significant human health effects. If the value of *CR* or *CCR* is between 10^−6^ and 10^−4^, it poses a carcinogenic risk, but this is acceptable. If the value of *CR* or *CCR* is ≥10^−4^, it indicates an unacceptable carcinogenic risk to human health [48]. The reference doses (RfD) and cancer slope factors (*CSF*) for the PTEs are shown in Table S3 [40,43,44,49,50].

### 2.5. Statistical Analysis

The Origin statistical package (version 9.8.0.200) was used to perform multivariate analysis including Pearson correlation analysis, hierarchical cluster analysis (HCA) and principal component analysis (PCA). IBM SPSS Statistics 23.0 software was used for descriptive data analysis. ArcGIS 10.3 software (Environmental Systems Research Institute, Inc. (ESRI)) was used to generate maps.

## 3. Results and Discussion

### 3.1. Content of PTEs in Soil

The statistical analysis results for the PTEs in the studied soils are summarized in Table 2, and the background values of PTEs in the soil of Gansu Province were compared and analysed. As is indicated in Table 2, each PTE exhibited an extremely wide distribution. The average concentration of each PTE decreased in the following order: As > Zn > Sb > Cu > Ni > Pb > Hg. Sb (33.34), Hg (0.84), Cu (30.42), As (116.40) and Zn (82.49) were present in higher concentrations than the background value in Gansu Province (Sb: 1.34; Hg: 0.03; Cu: 21.4; As: 11.1; Zn: 69.4). Therefore, Sb, Hg, Cu, As, and Zn may be influenced by external sources such as spill inputs, agricultural inputs, traffic inputs and other sources. In addition, the coefficient of variation (CV) was determined to indicate the degree of dispersion in the numerical fluctuations in PTE concentration. The CV values of the PTEs in the soil of the study area decreased in the following order: Sb > Hg > Pb > Zn > As > Ni > Cu. Sb, Hg, Pb and Zn had higher CV values in the study area. The CV values of Sb, Hg, Zn and Pb were relatively high (CV > 30%), indicating that human input played a major role with regard to these PTEs [51]. The CV values of Sb and Hg were extremely high (158 and 89%), and high Sb and Hg levels were detected in the spilled mine tailings (Table 2). This result suggests that the pollution degree of Sb and Hg in the soil may be caused by the leakage accident in this mining area. The CV value of the other metals was lower than 30%, which indicates that anthropogenic imports are low and probably come primarily from soil parent materials of natural sources [51].

Comparison with the secondary environmental quality standards for Chinese soils [52] revealed that most elements did not reach the critical values, with the exception of Sb, Hg and As. The concentrations of Cu and Zn in the soil exceeded the background values in Gansu Province and were both slightly higher than those in the mine tailings. In recent years, it has been reported that high Sb and As concentrations adversely affect crops and farmers, and long-term exposure to small amounts of PTEs, such as Cu, Ni and Zn, also cause serious health problems [14]. These elements tend to accumulate in the body through the food chain and can cause irreversible damage [53,54]. The mean concentrations of PTEs, *I_geo_*, EF and $E_{r}^{i}$ in the soils of this study are compared with other regions of the world in Table 3. The comparison shows that the average concentrations of As, Hg and Sb in the soils of Xihe County in this study area were higher than those reported in the East Attica region in Greece [55], the Jiadengyu Peatlands in the Altay Mountains in China [56] and the Al Uyaynah-Al Jubailah region in Saudi Arabia [57]. In contrast, the mean values of Pb concentrations in the soils in Xihe County were lower than the mean values reported in other regions. The mean values of Cu concentrations in Xihe County were higher than the mean values in the Al Uyaynah–Al Jubailah region, but lower than the mean values in the East Attica region and the Jiadengyu Peatlands in the Altay Mountains. In addition, the concentrations of Ni and Zn in Xihe County were both lower than those in the East Attica region, and they were higher than those in the Jiadengyu Peatlands in the Altay Mountains and the Al Uyaynah-Al Jubailah region.

### 3.2. Assessment of PTE Contamination

#### 3.2.1. Geo-Accumulation Index Analysis Results

The *I_geo_* values of various PTEs are shown in Figure S1. The *I_geo_* values of Cu, Ni, Pb and Zn were mostly below 0, and the contribution of natural source inputs to these PTEs is likely to be greater, according to the grading of the geo-accumulation index values. The *I_geo_* values of As, Hg and Sb were higher than 2 at most sampling sites, and the pollution level of these PTEs was 4 and above, according to the grading standard of *I_geo_* values, indicating moderate and higher levels of contamination. Among the studied PTEs, As, Hg and Sb cause more serious adverse effects on the ecological environment, among which Hg had the highest pollution level, reaching a heavy pollution level on average. Consequently, these three elements are the pollutants that should be considered for control. Comparison of the results with other areas in Table 3 showed that the degree of pollution with As, Hg and Sb was significantly higher than in the Peenya Industrial Area in India [58] and the Huangpi District in Wuhan, China [59], while all other PTEs produced less pollution than in other areas.

In general, these results indicate that the problem of soil pollution involving Sb, As and Hg is very serious in this area. Combined with the CV analysis results, which are obviously related to the degree of human interference, Ni, Pb, Zn and Cu occur in a critical state, with less human interference, and they may originate from natural sources.

#### 3.2.2. Enrichment Factor Analysis Results

*EF* can distinguish the source of PTEs and assess the degree of anthropogenic influence and can determine their degree of contamination [60]. An *EF* value lower than 2 indicates that a given PTE is derived exclusively from parent material or natural processes and that there is no or slight contamination [61]. *EF* values above 2 indicate the presence of above-moderate contamination and possible anthropogenic input of the element.

The *EF* values of the seven PTEs are provided in Table 3. The order of the average *EF* values of the potentially toxic elements are Hg (29.43) > Sb (25.21) > As (11.08) > Cu (1.49) > Zn (1.24) > Ni (1.05) > Pb (0.84). Therefore, Cu, Zn, Ni and Pb in the soil of the study area may be derived from natural processes and are not largely responsible for any contamination, while other potentially toxic elements may be more influenced by anthropogenic activities and produce heavy contamination. Among these PTEs, Sb, Hg and As were the obvious pollutants that caused notable or high levels of contamination, a result that is consistent with the geo-accumulation index. Comparison with the *EF* results of PTEs in the Al Uyaynah–Al Jubailah region, Saudi Arabia [57] and Huangpi District, Wuhan, China [59] revealed that As, Hg and Sb pollution in Xihe County was much higher than in other regions, and Pb pollution was slightly lower than in other regions. Cu and Ni pollution levels in Xihe County were higher than in the Al Uyaynah–Al Jubailah region and lower than in Huangpi District. Zn pollution was almost the same as in the Al Uyaynah–Al Jubailah region and both were higher than in Huangpi District.

#### 3.2.3. Results of *I_geo_* and *EF* Spatial Distribution of As, Hg and Sb

**Figure 2:**
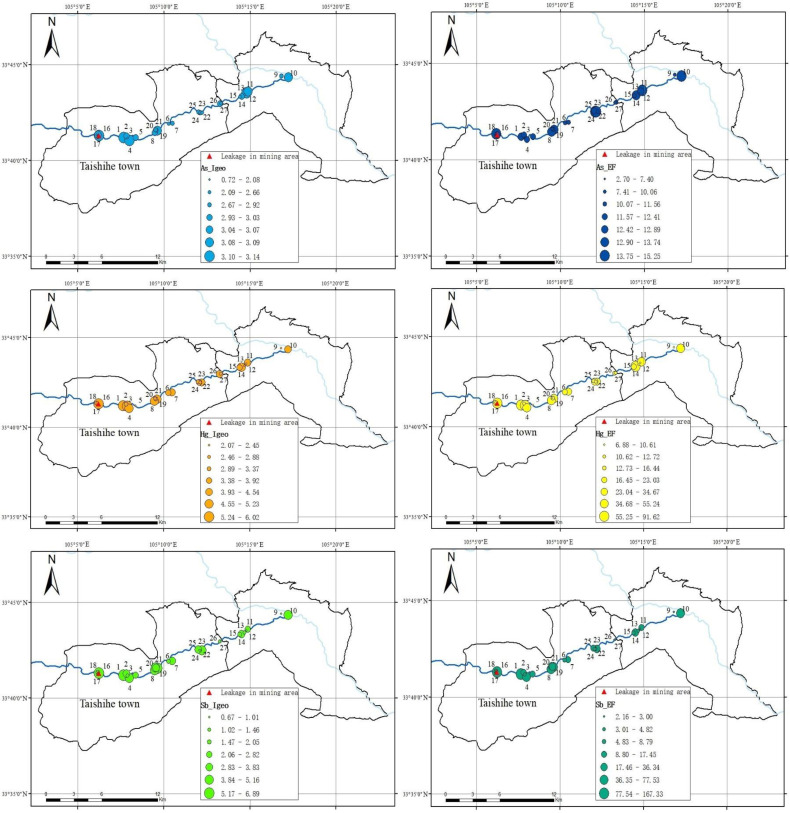
Spatial interpolation results of the content of heavily contaminated PTEs. The numbers next to the sampling locations indicate the numbers of the sampling points.

*I_geo_* and *EF* gradient distribution of PTEs with high pollution levels in soils were determined using a distribution model based on proportional/graded symbols to reflect the spatial variation in PTEs in soils [62]. In addition, the spatial distribution characteristics of As, Hg and Sb were analysed in this study (Figure 2). Figure 2 shows that the *I_geo_* and *EF* values of Sb and Hg presented the same spatial distribution pattern, both with two high levels in the southwestern and northeastern parts of the sampling area. Along the river to the east, the *I_geo_* and *EF* values gradually decreased, and a low-level area was present in the central area. This result is consistent with the flow direction of the river in the sampling area, suggesting that mine leakage is the main source of Sb and Hg, and that water flow is the main transportation method of Sb and Hg across the sampling area. To reduce PTE pollution in the lower reaches of the river, polluted river water was diverted, and sedimentation tanks were established in the northeast area of the sampling region to intercept the pollutants discharged from the tailings pond. Therefore, there were two high-level areas in the southwest and northeast, and the central area contained the lowest levels. This result is similar to the heavy metal pollution resulting from the Xikuangshan antimony mine [63].

The other PTE producing a high degree of contamination was As, whose *EF* maximum value did not occur at the spill site but downstream of it. The spatial distribution of *I_geo_* values for As was different from that of Sb and Hg. Its maximum values occurred near the centre of the sampling sites and its values were relatively low in the southwest and northeast. Consequently, it is obvious that the sources of As did not include mine leakage.

### 3.3. Ecological Risk Assessment

**Figure 3:**
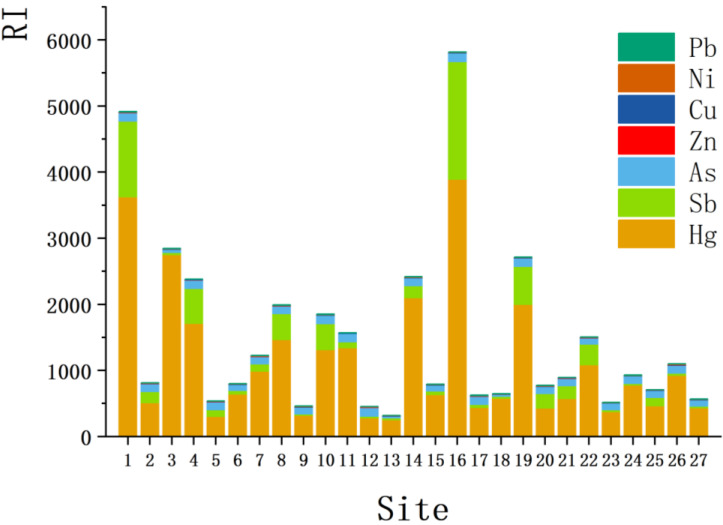
Ecological potential risk index of the various PTEs recorded in soils of the study area.

The results of the comprehensive potential ecological risk assessment of PTEs in soil and the potential ecological risk indices of individual elements are shown in Figure 3 and Table S4, respectively. Based on the $E_{r}^{i}$ values of individual elements, the elements with contamination levels from high to low were Hg (1118.87), Sb (248.84), As (104.86), Cu (7.11), Ni (5.00), Pb (3.95) and Zn (1.19). The *RI* values in the tailings spill area in this study ranged from 320.43 to 5820.46 (mean: 1489.82) and all sampling sites were contaminated to a considerable or higher degree, representing a very high potential ecological risk in the study area. According to Table 2 and Figure 3, Hg and Sb were found to yield certain risks. However, the high value of Hg differed from other PTEs due to its own high toxicity coefficient.

By comparing the potential ecological risks in Table 3 with those in the Al Uyaynah-Al Jubailah region of Saudi Arabia [57] and Huangpi District of Wuhan, China [59], it was found that the potential ecological risks of Hg, Sb and As in this study were much higher than those in other areas, causing very-high, high and considerable ecological risks, respectively. The ecological risk of Zn was also higher than in other areas; however, the risk was very low. In addition the ecological risk caused by Cu and Ni in Xihe County was higher than that of the Al Uyaynah-Al Jubailah region and lower than that of Huangpi District. Moreover, the ecological risk caused by Pb was lower than that of the other regions, all of which were low risk. The results indicate that the contamination of PTEs caused by the Sb mine leakage is more harmful than in other areas.

### 3.4. Source Identification of PTEs

#### 3.4.1. Correlation Analysis

Correlations between different PTEs are often adopted as a basis to determine whether the considered PTEs have a common source [64,65], and it is generally considered that PTEs with a significant positive correlation may exhibit similar sources.

The Pearson correlation coefficients between PTEs in the studied soils are listed in Table 4. At the *p* < 0.01 significance level, there were significantly positive correlations between Cu–Ni (0.68), Cu–Zn (0.51) and Ni–Zn (0.48), indicating that Cu, Ni and Zn may have had the same source. In addition, a significantly positive correlation was also observed between Sb and Hg (0.82), indicating that Sb and Hg were derived from a common pollution source, as the concentration of Sb far exceeded the background value. Positive correlations were found between As and Pb (0.39) at the significance level of *p* < 0.05. This suggests that As and Pb may have come from the same source.

#### 3.4.2. Principal Component Analysis

**Figure 4:**
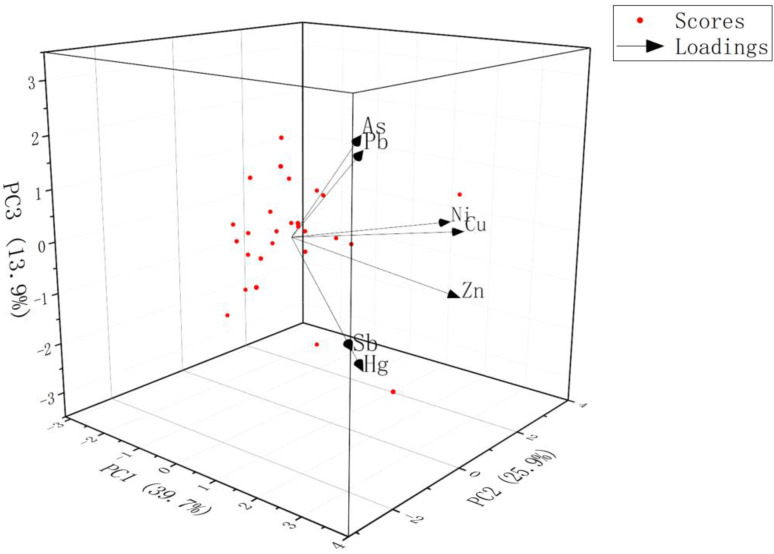
Three-dimensional principal component loading plot of the PCA results.

PCA has been applied to identify and qualitatively analyse the possible sources of potential toxic element contamination [66]. Applying PCA to the sample of this study (KMO = 0.589 and Barlett’s test (*p* < 0.001)) explained 79.40% of the total variance when three principal components were chosen. The results of PCA and the spatial variation in rotated principal components in this study are shown in Table S5 and Figure 4, respectively. In the first principal component (PC1), Zn, Sb and Hg accounted for a high loading, occupying 39.67% of the total variance. The second principal component (PC2) was able to explain 25.86% of the total variance, where Ni and Cu attained higher loads, and Zn attained a lower degree of loading. The third principal component (PC3) was able to explain 13.87% of the total variance under high loadings of As and Pb.

The first group of elements included Sb, Hg and Zn. The concentration of Sb and Hg far exceeded the background values, both of which were significantly correlated and pose a considerable risk to the ecological environment. It can be inferred that they both originated from the same source of pollution. The results of the geospatial analysis of Sb and Hg showed the same pattern and provided visualization of the impact of the spill. Obviously, Sb was mainly from this Sb mine spill, and Hg was equally from the tailings leaked in the accident. Previous studies have shown that non-ferrous metal smelting is the primary cause of Hg contamination in Chinese soils, accounting for 45% of the overall contamination level [67]. This could indicate that the presence of Sb mines in the study area could lead to contamination with Sb and Hg. In addition, high Hg levels may be associated with antimony mines [51]. The CV values for Sb and Hg also support that their contamination came from the influence of human activities. The mean Zn concentration in the soil was slightly higher than the background value; however, the Zn content in the tailings was lower, and was well below the background value and the soil concentration. It is unlikely that the tailings spill caused Zn concentrations to reach the current levels. Moreover, Zn levels were not affected by the accident in the same way as those of Sb and Hg, with dramatic fluctuations in concentrations, so Zn may not have come from the same source as Sb and Hg. Therefore, the first PC represents the tailings spill in the study area.

The concentration of the second group of PTEs, namely Cu, Ni and, to a lesser extent, Zn, were close to or slightly above the background values for soils in Gansu Province. The CV values of the coefficients of variation for Cu and Ni were lower and probably less subjected to anthropogenic interference. Moreover, according to the results of *I_geo_* and potential ecological risk index obtained above, Cu, Ni and Zn are less harmful to the ecological environment and, basically, do not cause ecological risk. Based on the results of the correlation analysis, there was a significant positive correlation between Cu, Ni and Zn. Moreover, the concentration of Zn was only slightly higher than the background value and did not increase substantially compared with the background value, and Cu, Ni and Zn may have come from the same source. Previous studies have shown that Ni is often associated with soil genesis processes and that the soil parent material is a major source of Ni [68]. The results above suggest that Cu, Ni and Zn may be from natural sources, mainly influenced by the natural geological background. Furthermore, a variety of metallic mineral resources are abundant in Xihe County; therefore, the origin is likely to be mainly from soil parent material and rock weathering processes.

The third group of elements consisted of As and Pb. The average concentration of As was much higher than the background value, and the pollution level and ecological risk caused by As were higher. The concentration of Pb was slightly lower than the background value (21.4 mg kg^−1^), and its corresponding impact was smaller. According to the results of the geospatial analysis of As, the pollution of As was more serious in the centre of the sampling area, which is the area with a dense distribution of towns and villages and a relatively developed population. It has been confirmed in many studies that herbicides and pesticides used in agricultural fields contain inorganic arsenic compounds [69], and the use of phosphate fertilizers is also a way for As to enter agricultural soils [70]. Therefore, As is often associated with agricultural activities. Some studies have shown that lead levels in roadside soils are associated with engine exhaust, tyre wear and brake lining wear, and that traffic emissions affect lead levels in roadside topsoil [71]. Yu et al. [72] found that vehicle traffic emissions from road dust collected in Harbin allowed Pb to be deposited in the soil, and that the prevailing local winds dictated that Pb was mainly distributed in the western and southern parts of the study area. Therefore, lead in agricultural soils is often derived from traffic emissions and atmospheric deposition, while the low Pb content in the study area excludes the possibility that the third PC was due to vehicle emissions. Therefore, As and Pb mainly originate from agricultural activities.

#### 3.4.3. Hierarchical Cluster Analysis

**Figure 5:**
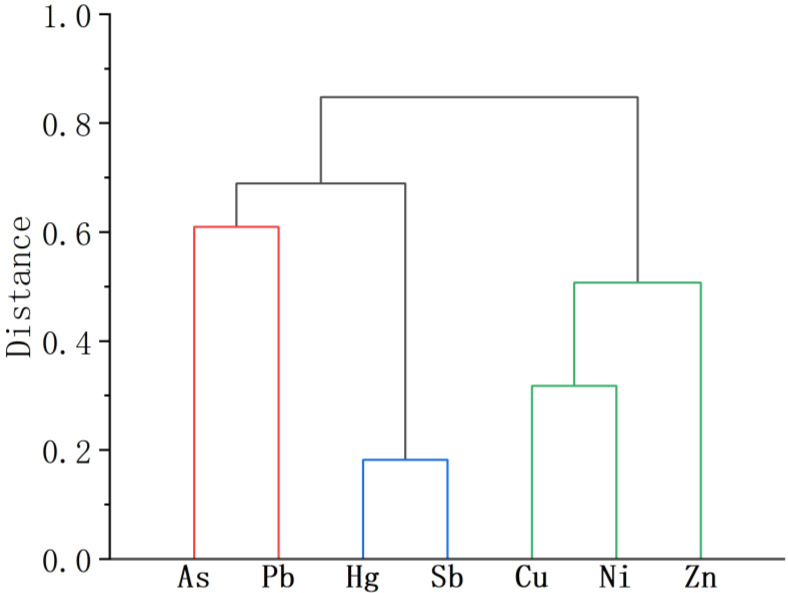
Dendrogram of HCA of the concentration of PTEs in the soil samples.

HCA is commonly used to analyse the sources of contamination of PTEs [10]. The results of HCA can reveal the relationships among the analysed elements, between and within groups. Elements from different sources are clustered according to the maximum and minimum levels of clustering similarity [32]. HCA can often be used as an auxiliary method to validate the results of PCA analysis and facilitate the classification of variables [73]. The results of HCA for the PTEs studied are shown in Figure 5. These elements can be classified into three groups: (1) Sb-Hg; (2) As-Pb; and (3) Cu-Ni-Zn. The HCA grouped Zn with Cu and Ni as natural sources, which were not classified as sources of antimony mine leakage, and this was also consistent with the results of the correlation analysis where Zn was most strongly correlated with Cu. The HCA results indicated that there were at least three sources of PTEs in the soil of this tailings leakage area. This was generally consistent with the PCA results.

### 3.5. Health Risk Assessment

#### 3.5.1. Non-Carcinogenic Risk

As and Sb accounted for the majority of non-carcinogenic risks caused by PTEs, and were the main elements posing a risk to health. The difference was that As presented a higher risk at most points, while Sb only presented a higher risk at points closer to the tailings spill. The health risk associated with Sb in the soil on both sides of the river section after dosing and dredging was subsequently reduced, proving that the emergency disposal measures were quite effective. This result also verified the previous conclusion that Sb came from the tailings leakage and As was mainly from agricultural activities.

**Figure 6:**
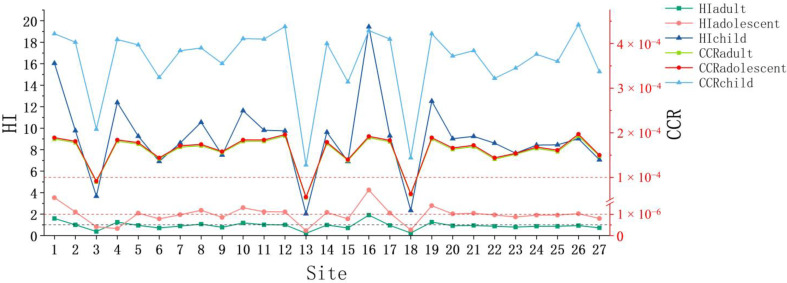
*HI* and *CCR* for the contamination of PTEs overall for adults, adolescents and children. The three reference lines in Figure 6 are, in order of meaning from bottom to top: the reference line for *HI* = 1, the reference line for *CCR* = 10^−6^ and the reference line for *CCR* = 10^−4^.

The hazard index and cumulative carcinogenic risk of three populations (adults, adolescents and children) exposed to PTEs at the 27 sampling sites were compared overall (Figure 6). The comparison revealed that children were exposed to the highest non-carcinogenic and carcinogenic risks among all populations, and that the risk values exceeded the standard limits at essentially all points, which may negatively affect their health. Children are the most vulnerable and susceptible group in environments contaminated by PTEs, primarily because of their small size, low weight, incomplete physiological development and greater exposure to soil than adults and adolescents [74]. The health risks to children from the antimony mine tailings spill are difficult to ignore and may have a significant impact. Consequently, it is important to control the amount of time children spend outdoors until the ecological conditions along the Taishi River are fully restored.

**Figure 7:**
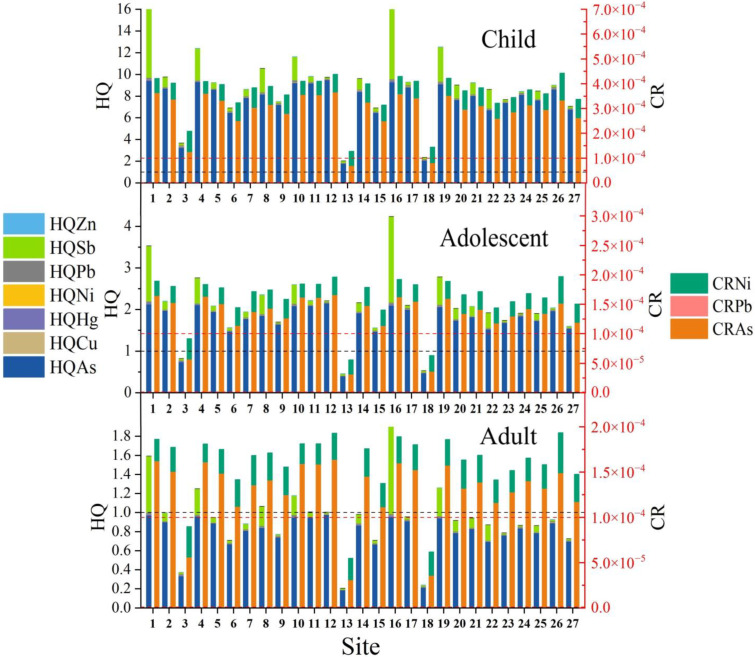
Non-carcinogenic and carcinogenic risks of different PTEs for three populations. The black reference line in Figure 7 is the reference line for *HQ* = 1 and the red reference line is the reference line for *CCR* = 10^−4^.

Analysis of the non-carcinogenic risk results clearly revealed that, of the three exposure routes, the highest risk was associated with soil ingestion, followed by dermal exposure, and the lowest risk was due to the inhalation of soil (Table S6). As can be seen in Figure 7, children and adolescents exceeded the non-carcinogenic risk limit at almost all sites. In contrast, adults were exposed to higher non-carcinogenic risks mainly in areas closer to the tailings spill, and risk values exceeded the limit at only four sites. This indicates that the main populations affected by the tailings spill were children and adolescents, with adults being exposed to some non-carcinogenic risks in individual areas.

#### 3.5.2. Carcinogenic Risk

Children have the highest cumulative carcinogenic risk. Adolescents and adults have roughly equal cumulative carcinogenic risk, with adolescents having a slightly higher risk than adults. However, all three populations have carcinogenic risk values exceeding 10^−4^ at all points, and PTEs present a carcinogenic risk to residents. The PTEs that cause cancer include As, Ni and Pb, with the highest carcinogenic risk being associated with As. For adults, As poses a great risk to human health that cannot be ignored, Ni is associated with a cancer risk value between 10^−6^ and 10^−4^, which is significant but tolerable, and Pb is associated with a cancer risk value below 10^−6^ that can be ignored. Adolescents and children are exposed to the same carcinogenic risks as adults.

The non-carcinogenic risk is the highest for children. The carcinogenic risk for adolescents and adults is approximately the same. The carcinogenic risk for adolescents is slightly higher than that for adults. However, the carcinogenic risk for all three groups at each point is more than 10^−4^. 

## 4. Conclusions

This study revealed potential ecological and health risks, and sources of pollution caused by the antimony mine spill, and produced the following key conclusions:

1. The concentration of Sb and Hg in the study area were relatively excessive due to the antimony mine tailings spill.
2. As, Hg and Sb were responsible for high levels of ecological contamination and they are associated with potential ecological risk.
3. The multivariate statistical analysis showed that the areas highly contaminated with Hg and Sb were mainly due to the tailings spill. As in the soil was mainly from agricultural activities.
4. The results of the spatial distribution revealed that Sb and Hg showed a trend of high levels in the southwest and northeast of the sampling area, a pattern consistent with these levels being due to the tailings leakage. The levels of As were higher in the dense urban regions in the central part of the study area, associated with anthropogenic activities. This corroborates the conclusions of the multivariate statistical analysis.
5. The non-carcinogenic or carcinogenic risk is too high in all populations except for the non-carcinogenic risk for adults. Of the three population groups, children are the most vulnerable group. Ingestion is the most significant exposure pathway for potentially toxic elements posing a threat to human health. Non-carcinogenic risk from Sb and non-carcinogenic and carcinogenic risks from As are the main contributors to the health risk. Furthermore, the risks associated with Sb and As at the sampling sites were verified and Sb originated from antimony mine spills and As originated from agricultural activities.

This study is important because it contributes to the understanding of the ecological risks associated with antimony mine spills and the potential sources of contamination from such events. The results of this study can inform the development of effective remediation strategies to mitigate the environmental impacts of mine spills. The future scope of this study includes further investigation of the ecological risks and sources of contamination from antimony and other heavy metal mines. Future research could build on the results of this study to further investigate the potential sources of contamination and develop more effective remediation strategies.

Overall, the contribution of this study to the national and international research community is that it expands the understanding of the ecological risks posed by antimony mine spills and the sources of contamination from such events. The results of the study can provide a scientific basis for future research and guide managers in developing effective remediation measures. Furthermore, this study can provide a reference for risk assessment at other mine sites around the world where tailings spills have occurred.

## Figures and Tables

**Table 1:** Geo-accumulation index [27], enrichment factor [29], individual potential ecological risk factor $E_{r}^{i}$ and comprehensive potential ecological risk index (*RI*) [30] level criteria classification.

Enrichment Factor (EF)	Degree of Pollution	Geo-Accumulation Index (*I_geo_*)	Degree of Pollution	Individual Potential Ecological $\mathrm{Risk}\;\mathrm{Factor}\;E_{r}^{i}$	Degree of Pollution	Comprehensive Potential Ecological Risk Index (*RI*)	Degree of Pollution
EF < 2	No or minimal pollution	*I_geo_* < 0	Practically uncontaminated	$E_{r}^{i}$ < 40	Low risk	*RI* < 150	Low risk
2 ≤ EF < 5	Moderate pollution	0 ≤ *I_geo_* < 1	Uncontaminated to moderately contaminated	40 ≤ $E_{r}^{i}$ < 80	Moderate risk	150 ≤ *RI* < 300	Moderate risk
5 ≤ EF < 20	Notable pollution	1 ≤ *I_geo_* < 2	Moderately contaminated	80 ≤ $E_{r}^{i}$ < 160	Considerable risk	300 ≤ *RI* < 600	Considerable risk
20 ≤ EF < 40	High pollution	2 ≤ *I_geo_* < 3	Moderately to heavily contaminated	160 ≤ $E_{r}^{i}$ < 320	High risk	*RI* ≥ 600	High risk
EF ≥ 40	Extreme pollution	3 ≤ *I_geo_* < 4	Heavily contaminated	$E_{r}^{i}$ ≥ 320	Very-high risk		
		4 ≤ *I_geo_* < 5	Heavily to extremely contaminated				
		*I_geo_* ≥ 5	Extremely contaminated				

**Table 2:** Descriptive statistical summary of the potentially toxic element (PTE) concentration (mg kg^−1^) in the studied soil.

Values	As	Cu	Hg	Ni	Pb	Sb	Zn	Al
Maximum	146.28	44.7	2.92	45.3	33.2	238.2	176.2	99,096
Minimum	27.46	24.4	0.19	20.6	7.2	3.2	55.3	50,774.2
Mean	116.40	30.42	0.84	28.78	16.89	33.34	82.49	65,253.93
SD	32.28	4.90	0.75	4.79	6.51	52.64	28.83	9347.84
CV	0.28	0.16	0.89	0.17	0.39	1.58	0.35	0.14
Gansu ^a^	11.1	21.4	0.03	28.8	21.4	1.34	69.4	68,300
Tailing ^b^	156.6	14.9	7.85	–	112.1	2493.3	38.7	–
Grade II	20–25	100	0.5–1.0	50–60	300–350	–	250–300	–

–, Not available. ^a^. Background values in Gansu Province. ^b^. Metal levels in mining tailings.

**Table 3:** Comparison of PTE concentrations, *I_geo_*, *EF* and $E_{r}^{i}$ in soil from different regions of the world.

Region	Index	As	Cu	Hg	Ni	Pb	Sb	Zn	Reference
Xihe County, China	Concentration (mg kg^−1^)	116.40	30.42	0.84	28.78	16.89	33.34	82.49	This study
The East Attica region, Greece		41.8	31.9	–	172	217	3.5	170	[55]
The Jiadengyu Peatlands in the Altay Mountains, China		3.75	37.60	0.26	19.62	25.57	0.67	46.17	[56]
Al Uyaynah–Al Jubailah region, Saudi Arabia		13.8	10.56	0.11	19.25	28.48	0.067	64.33	[57]
Xihe County, China	*I_geo_*	2.71	-0.09	3.76	−0.60	−1.03	2.84	−0.40	This study
The Peenya Industrial Area, Bengarulu, India		−0.05	9.39	–	–	8.77	− 0.57	12	[58]
Huangpi district, Wuhan, China		−0.53	0.21	−0.58	0.99	0.5	−2.09	−5.5	[59]
Xihe County, China	*EF*	11.08	1.49	29.43	1.05	0.84	25.21	1.24	This study
Al Uyaynah–Al Jubailah region, Saudi Arabia		2.79	0.37	2.11	0.91	1.47	1.49	1.25	[57]
Huangpi district, Wuhan, China		1.36	2.13	2.02	3.44	2.99	0.38	0.04	[59]
Xihe County, China	$E_{r}^{i}$	104.86	7.11	1118.87	5.00	3.95	248.84	1.19	This study
Al Uyaynah–Al Jubailah region, Saudi Arabia		20.20	1.36	62.29	3.28	5.27	0.50	0.90	[57]
Huangpi district, Wuhan, China		10.39	10.39	40	14.89	10.59	5.27	0.03	[59]

**Table 4:** Pearson correlation matrix for the PTEs in the soil samples.

	As	Cu	Hg	Ni	Pb	Sb	Zn
**As**	1	0.21	0.21	−0.23	0.39 *	0.37	0.27
**Cu**		1	0.14	0.68 **	0.10	0.07	0.51 **
**Hg**			1	−0.02	0.31	0.82 **	0.41 *
**Ni**				1	0.13	−0.16	0.48 *
**Pb**					1	0.35	0.23
**Sb**						1	0.47 *
**Zn**							1

* The correlation is significant at the 0.05 level (two-tailed). ** The correlation is significant at the 0.01 level (two-tailed).

## Data Availability

Data are contained within the article or the supplementary materials.

## Supplementary Materials

The following supporting information can be downloaded at: https://www.mdpi.com/article/10.3390/toxics11040359/s1, Figure S1: Geological accumulation index of the various PTEs; Table S1: Reference material for quality control; Table S2: Human activity parameters; Table S3: Reference dose (RfD) (mg kg^−1^ d^−1^) and cancer slope factor (CSF) ((mg kg^−1^ d^−1^)^−1^) of potentially toxic elements; Table S4: The results of the comprehensive potential ecological risk assessment of PTEs in soil and the potential ecological risk indices of individual elements; Table S5: Principal component analysis results for the tested soils; Table S6: Non-carcinogenic risk results for adults contaminated with PTEs. 1, 2 and 3 represent the different routes of exposure: 1 for ingestion, 2 for inhalation and 3 for dermal contact.
