# Supplementary material for: Potentially Toxic Element Contamination in Soils Affected by the Antimony Mine Spill in Northwest China
Source: Toxics. 2023 Apr 10;11(4):359. doi: 10.3390/toxics11040359 (PMC10140868; doi:10.3390/toxics11040359)
Supplement: Supplementary file 1 [file toxics-11-00359-s001.zip › toxics-2311691-supplementary.pdf]

toxics

Article

Potentially Toxic Element Contamination in Soils Affected by the Antimony Mine Spill in Northwest China

Yongzhen Chai and Fei Guo

Supplementary Materials

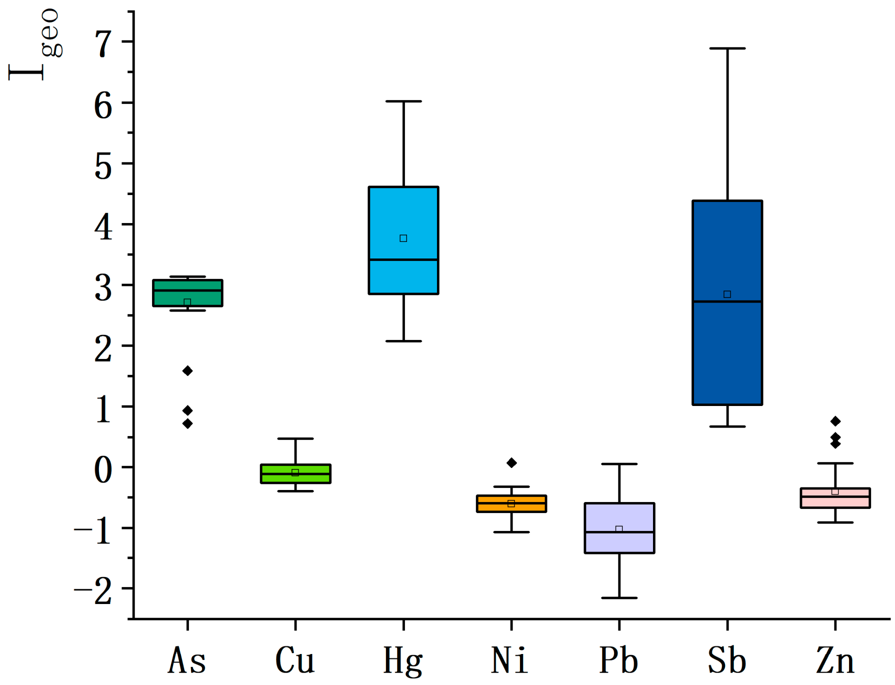

Figure S1. Geo-logical accumulation index of the various PTEs.

Table S1. Reference material for quality control.

PTEs	Reference Material Product Number	Concentration (μg/mL)
As	GBW08611	1000
Cu	GBW08615	1000
Hg	GBW08617	1000
Ni	GBW08618	1000
Pb	GBW08619	1000
Sb	GBW(E)080295	1000
Zn	GBW08620	1000

Table S2. Human activity parameters.

	Child	Teenager	Adult	Description	References
IngR **(mg d^{-1})**	200	150	100	Ingestion Rate of Soils	[40]
InhR **(m^3 day^{-1})**	7.5	11.25	15	Inhalation Rate of Soil	[40]
ED **(year)**	6	12	26	Exposure Duration	[40–42]
EF **(d year^{-1})**	350	350	350	Exposure Frequency	[40]
SA **(cm^2)**	2373	4036.5	5700	Exposured Skin Area	[40]
ABS	0.001 except for As (0.03)	0.001 except for As (0.03)	0.001 except for As (0.03)	Dermal Absorption Factor	[43,44]
AF **(mg cm^{-2})**	0.2	0.135	0.07	Adherence Factor	[40]
PEF **(m^3 kg^{-1})**	1.36×10^9	1.36×10^9	0.000296	Particle Emission Factor	[45]
BW **(kg)**	11.58	41.33	61.8	Average Body Weight	[41,42]
AT **(d)**	25550<br>365 × ED=2190	25550<br>365 × ED=4380	25550<br>365 × ED=9490	Average Time for Carcinogenic (Non-Carcinogenic) Effect	[40]

Table S3. Reference dose (RfD) (mg kg^{-1} d^{-1}) and cancer slope factor (CSF) ((mg kg^{-1} d^{-1})$^{-1}$) of potentially toxic elements.

	As	Cu	Hg	Ni	Pb	Sb	Zn
RfD for Ingestion	3.00×10^{-4}	4.00×10^{-2}	3.00×10^{-4}	2.00×10^{-2}	3.00×10^{-4}	4.00×10^{-4}	3.00×10^{-1}
RfD for Dermal Absorption	1.23×10^{-4}	1.20×10^{-2}	2.10×10^{-5}	5.40×10^{-3}	2.10×10^{-5}	-	6.00×10^{-2}
RfD for Inhalation	3.00×10^{-4}	4.02×10^{-2}	8.57×10^{-5}	2.06×10^{-2}	8.57×10^{-5}	-	3.00×10^{-1}
SF for Ingestion	1.50	-	-	1.7	0.0085	-	-
SF for Dermal Absorption	3.66	-	-	-	-	-	-
SF for Inhalation	15.1	-	-	8.40×10^{-1}	0.042	-	-

Date from [40,43,44,49,50].

Table S4. The results of the comprehensive potential ecological risk assessment of PTEs in soil and the potential ecological risk indexes of individual elements.

Site	As	Cu	Hg	Ni	Pb	Sb	Zn	RI	Risk level
S1	303.72	31.98	2.44	2810.87	1.51	3.33	1.11	3156.72	High
S2	44.66	29.7	3.03	395.15	1.71	1.49	0.85	478.33	Considerable
S3	9.33	11.04	3.06	2131.15	2.13	1.57	1.37	2161.18	High
S4	139.95	31.72	3.43	1326.59	1.27	1.42	0.9	1507.12	High
S5	26.4	29.31	2.96	236.32	1.67	1.03	1.96	301.41	Considerable
S6	14.49	22.07	2.7	495.9	1.89	1.57	1.02	541.09	Considerable
S7	29.78	26.7	3.27	765.18	2.09	1.84	1.57	832.21	High
S8	105.01	27.8	2.97	1135.84	1.92	2.51	1.08	1278.64	High
S9	6.55	24.56	3.46	246.66	1.97	2.52	1.07	288.56	Moderate
S10	104.22	31.36	2.84	1017.21	1.41	4.13	1.48	1164.1	High
S11	23.42	31.3	2.63	1040.05	1.42	1.14	0.94	1102.37	High
S12	6.75	32.29	2.99	219.27	1.85	1.9	1.04	267.87	Moderate
S13	8.14	6.06	2.65	195.4	1.51	0.89	0.84	217.08	Moderate
S14	48.04	28.63	4.12	1628.33	1.94	1.99	1.18	1715.69	High
S15	14.69	21.97	3.14	488.97	1.66	1.96	1.02	534.89	Considerable
S16	472.84	31.55	3.51	3017.23	1.84	2.65	2.54	3534	High
S17	13.1	30.05	2.75	339.04	1.77	3.59	1.07	392.91	Considerable
S18	7.15	7.02	2.68	449.4	1.65	1.9	0.91	472.28	Considerable
S19	152.26	31.01	2.99	1549.38	1.81	2.92	1.1	1743.12	High
S20	58.56	26.03	3.58	332.84	1.98	2.51	1.05	428.41	Considerable
S21	51.81	27.36	3.3	444.02	1.89	1.45	1.15	532.87	Considerable
S22	83.57	22.88	2.75	840.93	1.61	1.74	0.8	955.96	High
S23	7.54	25.2	2.45	290.16	1.53	1.55	0.92	330.64	Considerable
S24	6.35	27.64	2.51	603.36	1.6	2.93	0.96	646.7	High

S25	33.94	26.01	2.51	358.57	1.67	1.4	0.98	426.63	Considerable
S26	8.34	29.39	4.47	717.75	2.8	2.82	2.12	770.21	High
S27	6.55	23.09	2.96	336.45	1.93	1.9	1.11	375.54	Considerable
Mean	104.87	7.11	1118.87	5.00	3.95	248.84	1.19	1489.82	High

Table S5. Principal component analysis results for the tested soils.

Element	PC1	PC2	PC3
As	0.33	−0.15	0.64
Cu	0.33	0.51	−0.01
Hg	0.44	−0.31	−0.40
Ni	0.23	0.63	-0.01
Pb	0.33	−0.14	0.57
Sb	0.45	−0.40	−0.26
Zn	0.47	0.21	−0.21
Eigenvalues	2.78	1.81	0.97
Variance Contribution Rate/%	39.67	25.86	13.87
Cumulative Variance Contribution Rate/%	39.67	65.53	79.40

Table S6. Non-carcinogenic risk results for adults contaminated with PTEs. 1,2,3 represent the different routes of exposure, 1 for ingestion, 2 for inhalation, and 3 for dermal contact.

Site	HQAs1	HQAs2	HQAs3	HQCu1	HQCu2	HQCu3	HQHg1	HQHg2	HQHg3
1	8.00	2.21×10^{-4}	2.19×10^{-1}	1.01×10^{-2}	2.77×10^{-7}	7.99×10^{-5}	1.50×10^{-1}	1.45×10^{-5}	5.09×10^{-3}
2	7.43	2.05×10^{-4}	2.03×10^{-1}	1.25×10^{-2}	3.44×10^{-7}	9.92×10^{-5}	2.11×10^{-2}	2.04×10^{-6}	7.16×10^{-4}
3	2.76	7.61×10^{-5}	7.55×10^{-2}	1.27×10^{-2}	3.48×10^{-7}	1.00×10^{-4}	1.14×10^{-1}	1.10×10^{-5}	3.86×10^{-3}
4	7.93	2.19×10^{-4}	2.17×10^{-1}	1.42×10^{-2}	3.90×10^{-7}	1.12×10^{-4}	7.09×10^{-2}	6.84×10^{-6}	2.40×10^{-3}
5	7.33	2.02×10^{-4}	2.00×10^{-1}	1.23×10^{-2}	3.36×10^{-7}	9.69×10^{-5}	1.26×10^{-2}	1.22×10^{-6}	4.28×10^{-4}
6	5.52	1.52×10^{-4}	1.51×10^{-1}	1.12×10^{-2}	3.07×10^{-7}	8.84×10^{-5}	2.65×10^{-2}	2.56×10^{-6}	8.98×10^{-4}
7	6.68	1.84×10^{-4}	1.83×10^{-1}	1.35×10^{-2}	3.71×10^{-7}	1.07×10^{-4}	4.09×10^{-2}	3.95×10^{-6}	1.39×10^{-3}
8	6.95	1.92×10^{-4}	1.90×10^{-1}	1.23×10^{-2}	3.37×10^{-7}	9.73×10^{-5}	6.07×10^{-2}	5.86×10^{-6}	2.06×10^{-3}
9	6.14	1.69×10^{-4}	1.68×10^{-1}	1.43×10^{-2}	3.93×10^{-7}	1.13×10^{-4}	1.32×10^{-2}	1.27×10^{-6}	4.47×10^{-4}
10	7.84	2.16×10^{-4}	2.15×10^{-1}	1.18×10^{-2}	3.23×10^{-7}	9.30×10^{-5}	5.43×10^{-2}	5.25×10^{-6}	1.84×10^{-3}
11	7.83	2.16×10^{-4}	2.14×10^{-1}	1.09×10^{-2}	2.98×10^{-7}	8.60×10^{-5}	5.56×10^{-2}	5.36×10^{-6}	1.88×10^{-3}
12	8.08	2.23×10^{-4}	2.21×10^{-1}	1.24×10^{-2}	3.40×10^{-7}	9.79×10^{-5}	1.17×10^{-2}	1.13×10^{-6}	3.97×10^{-4}
13	1.52	4.18×10^{-5}	4.15×10^{-2}	1.10×10^{-2}	3.01×10^{-7}	8.68×10^{-5}	1.04×10^{-2}	1.01×10^{-6}	3.54×10^{-4}
14	7.16	1.97×10^{-4}	1.96×10^{-1}	1.71×10^{-2}	4.68×10^{-7}	1.35×10^{-4}	8.70×10^{-2}	8.40×10^{-6}	2.95×10^{-3}
15	5.50	1.52×10^{-4}	1.50×10^{-1}	1.30×10^{-2}	3.57×10^{-7}	1.03×10^{-4}	2.61×10^{-2}	2.52×10^{-6}	8.86×10^{-4}
16	7.89	2.18×10^{-4}	2.16×10^{-1}	1.45×10^{-2}	3.99×10^{-7}	1.15×10^{-4}	1.61×10^{-1}	1.56×10^{-5}	5.46×10^{-3}
17	7.52	2.07×10^{-4}	2.06×10^{-1}	1.14×10^{-2}	3.12×10^{-7}	9.01×10^{-5}	1.81×10^{-2}	1.75×10^{-6}	6.14×10^{-4}
18	1.76	4.84×10^{-5}	4.80×10^{-2}	1.11×10^{-2}	3.04×10^{-7}	8.78×10^{-5}	2.40×10^{-2}	2.32×10^{-6}	8.14×10^{-4}
19	7.76	2.14×10^{-4}	2.12×10^{-1}	1.24×10^{-2}	3.40×10^{-7}	9.79×10^{-5}	8.28×10^{-2}	7.99×10^{-6}	2.81×10^{-3}
20	6.51	1.79×10^{-4}	1.78×10^{-1}	1.48×10^{-2}	4.07×10^{-7}	1.17×10^{-4}	1.78×10^{-2}	1.72×10^{-6}	6.03×10^{-4}
21	6.84	1.89×10^{-4}	1.87×10^{-1}	1.37×10^{-2}	3.75×10^{-7}	1.08×10^{-4}	2.37×10^{-2}	2.29×10^{-6}	8.05×10^{-4}
22	5.72	1.58×10^{-4}	1.57×10^{-1}	1.14×10^{-2}	3.12×10^{-7}	9.01×10^{-5}	4.49×10^{-2}	4.34×10^{-6}	1.52×10^{-3}
23	6.30	1.74×10^{-4}	1.72×10^{-1}	1.01×10^{-2}	2.78×10^{-7}	8.02×10^{-5}	1.55×10^{-2}	1.50×10^{-6}	5.25×10^{-4}
24	6.91	1.91×10^{-4}	1.89×10^{-1}	1.04×10^{-2}	2.85×10^{-7}	8.22×10^{-5}	3.22×10^{-2}	3.11×10^{-6}	1.09×10^{-3}
25	6.50	1.79×10^{-4}	1.78×10^{-1}	1.04×10^{-2}	2.85×10^{-7}	8.22×10^{-5}	1.92×10^{-2}	1.85×10^{-6}	6.49×10^{-4}
26	7.35	2.03×10^{-4}	2.01×10^{-1}	1.85×10^{-2}	5.08×10^{-7}	1.46×10^{-4}	3.83×10^{-2}	3.70×10^{-6}	1.30×10^{-3}
27	5.78	1.59×10^{-4}	1.58×10^{-1}	1.23×10^{-2}	3.36×10^{-7}	9.69×10^{-5}	1.80×10^{-2}	1.73×10^{-6}	6.09×10^{-4}

Site	HQNi1	HQNi2	HQNi3	HQPb1	HQPb2	HQPb3	HQSb1	HQZn1	HQZn2	HQZn3
1	2.03×10^{-2}	5.43×10^{-7}	1.78×10^{-4}	1.27×10^{-1}	3.50×10^{-6}	2.01×10^{-3}	6.33	4.25×10^{-3}	1.17×10^{-7}	5.04×10^{-5}

2	2.29×10^{-2}	6.12×10^{-7}	2.01×10^{-4}	5.68×10^{-2}	1.57×10^{-6}	8.98×10^{-4}	9.32×10^{-1}	3.27×10^{-3}	9.03×10^{-8}	3.88×10^{-5}
3	2.86×10^{-2}	7.65×10^{-7}	2.51×10^{-4}	5.96×10^{-2}	1.64×10^{-6}	9.43×10^{-4}	1.95×10^{-1}	5.24×10^{-3}	1.45×10^{-7}	6.22×10^{-5}
4	1.71×10^{-2}	4.57×10^{-7}	1.50×10^{-4}	5.39×10^{-2}	1.49×10^{-6}	8.53×10^{-4}	2.92	3.44×10^{-3}	9.48×10^{-8}	4.08×10^{-5}
5	2.24×10^{-2}	6.01×10^{-7}	1.97×10^{-4}	3.93×10^{-2}	1.08×10^{-6}	6.21×10^{-4}	5.51×10^{-1}	7.51×10^{-3}	2.07×10^{-7}	8.91×10^{-5}
6	2.53×10^{-2}	6.76×10^{-7}	2.22×10^{-4}	5.96×10^{-2}	1.64×10^{-6}	9.43×10^{-4}	3.02×10^{-1}	3.90×10^{-3}	1.07×10^{-7}	4.62×10^{-5}
7	2.79×10^{-2}	7.48×10^{-7}	2.46×10^{-4}	7.00×10^{-2}	1.93×10^{-6}	1.11×10^{-3}	6.21×10^{-1}	6.01×10^{-3}	1.66×10^{-7}	7.13×10^{-5}
8	2.57×10^{-2}	6.87×10^{-7}	2.26×10^{-4}	9.56×10^{-2}	2.64×10^{-6}	1.51×10^{-3}	2.19	4.13×10^{-3}	1.14×10^{-7}	4.90×10^{-5}
9	2.63×10^{-2}	7.05×10^{-7}	2.31×10^{-4}	9.61×10^{-2}	2.65×10^{-6}	1.52×10^{-3}	1.37×10^{-1}	4.10×10^{-3}	1.13×10^{-7}	4.86×10^{-5}
10	1.89×10^{-2}	5.05×10^{-7}	1.66×10^{-4}	1.57×10^{-1}	4.33×10^{-6}	2.49×10^{-3}	2.17	5.65×10^{-3}	1.56×10^{-7}	6.71×10^{-5}
11	1.91×10^{-2}	5.11×10^{-7}	1.68×10^{-4}	4.33×10^{-2}	1.19×10^{-6}	6.85×10^{-4}	4.89×10^{-1}	3.61×10^{-3}	9.96×10^{-8}	4.29×10^{-5}
12	2.48×10^{-2}	6.65×10^{-7}	2.18×10^{-4}	7.24×10^{-2}	2.00×10^{-6}	1.15×10^{-3}	1.41×10^{-1}	3.97×10^{-3}	1.09×10^{-7}	4.71×10^{-5}
13	2.03×10^{-2}	5.43×10^{-7}	1.78×10^{-4}	3.41×10^{-2}	9.39×10^{-7}	5.39×10^{-4}	1.70×10^{-1}	3.20×10^{-3}	8.83×10^{-8}	3.80×10^{-5}
14	2.60×10^{-2}	6.96×10^{-7}	2.29×10^{-4}	7.57×10^{-2}	2.09×10^{-6}	1.20×10^{-3}	1.00	4.50×10^{-3}	1.24×10^{-7}	5.34×10^{-5}
15	2.22×10^{-2}	5.94×10^{-7}	1.95×10^{-4}	7.48×10^{-2}	2.06×10^{-6}	1.18×10^{-3}	3.06×10^{-1}	3.91×10^{-3}	1.08×10^{-7}	4.64×10^{-5}
16	2.47×10^{-2}	6.61×10^{-7}	2.17×10^{-4}	1.01×10^{-1}	2.78×10^{-6}	1.59×10^{-3}	9.86	9.73×10^{-3}	2.68×10^{-7}	1.15×10^{-4}
17	2.37×10^{-2}	6.34×10^{-7}	2.08×10^{-4}	1.37×10^{-1}	3.77×10^{-6}	2.16×10^{-3}	2.73×10^{-1}	4.10×10^{-3}	1.13×10^{-7}	4.87×10^{-5}
18	2.21×10^{-2}	5.92×10^{-7}	1.94×10^{-4}	7.24×10^{-2}	2.00×10^{-6}	1.15×10^{-3}	1.49×10^{-1}	3.47×10^{-3}	9.57×10^{-8}	4.12×10^{-5}
19	2.43×10^{-2}	6.50×10^{-7}	2.13×10^{-4}	1.11×10^{-1}	3.07×10^{-6}	1.76×10^{-3}	3.18	4.21×10^{-3}	1.16×10^{-7}	5.00×10^{-5}
20	2.65×10^{-2}	7.09×10^{-7}	2.33×10^{-4}	9.56×10^{-2}	2.64×10^{-6}	1.51×10^{-3}	1.22	4.01×10^{-3}	1.11×10^{-7}	4.76×10^{-5}
21	2.53×10^{-2}	6.78×10^{-7}	2.23×10^{-4}	5.54×10^{-2}	1.53×10^{-6}	8.76×10^{-4}	1.08	4.41×10^{-3}	1.21×10^{-7}	5.23×10^{-5}
22	2.15×10^{-2}	5.76×10^{-7}	1.89×10^{-4}	6.62×10^{-2}	1.83×10^{-6}	1.05×10^{-3}	1.74	3.05×10^{-3}	8.42×10^{-8}	3.62×10^{-5}
23	2.05×10^{-2}	5.48×10^{-7}	1.80×10^{-4}	5.91×10^{-2}	1.63×10^{-6}	9.36×10^{-4}	1.57×10^{-1}	3.53×10^{-3}	9.73×10^{-8}	4.19×10^{-5}
24	2.14×10^{-2}	5.74×10^{-7}	1.88×10^{-4}	1.12×10^{-1}	3.08×10^{-6}	1.77×10^{-3}	1.32×10^{-1}	3.67×10^{-3}	1.01×10^{-7}	4.35×10^{-5}
25	2.24×10^{-2}	5.99×10^{-7}	1.97×10^{-4}	5.35×10^{-2}	1.47×10^{-6}	8.46×10^{-4}	7.08×10^{-1}	3.74×10^{-3}	1.03×10^{-7}	4.44×10^{-5}
26	3.75×10^{-2}	1.00×10^{-6}	3.30×10^{-4}	1.07×10^{-1}	2.96×10^{-6}	1.70×10^{-3}	1.74×10^{-1}	8.11×10^{-3}	2.24×10^{-7}	9.62×10^{-5}
27	2.58×10^{-2}	6.92×10^{-7}	2.27×10^{-4}	7.24×10^{-2}	2.00×10^{-6}	1.15×10^{-3}	1.37×10^{-1}	4.23×10^{-3}	1.17×10^{-7}	5.02×10^{-5}

(continued from the above table)
